# Deciphering the Roles of PPARγ in Adipocytes via Dynamic Change of Transcription Complex

**DOI:** 10.3389/fendo.2018.00473

**Published:** 2018-08-21

**Authors:** Xinran Ma, Dongmei Wang, Wenjun Zhao, Lingyan Xu

**Affiliations:** Shanghai Key Laboratory of Regulatory Biology, Institute of Biomedical Sciences and School of Life Sciences, East China Normal University, Shanghai, China

**Keywords:** PPAR gamma, adipocytes, transcription complex, obesity, metabolic diseases

## Abstract

Peroxisome proliferator-activated receptor γ (PPARγ), a ligand-dependent transcription factor highly expressed in adipocytes, is a master regulator of adipogenesis and lipid storage, a central player in thermogenesis and an active modulator of lipid metabolism and insulin sensitivity. As a nuclear receptor governing numerous target genes, its specific signaling transduction relies on elegant transcriptional and post-translational regulations. Notably, in response to different metabolic stimuli, PPARγ recruits various cofactors and forms distinct transcriptional complexes that change dynamically in components and epigenetic modification to ensure specific signal transduction. Clinically, PPARγ activation via its full agonists, thiazolidinediones, has been shown to improve insulin sensitivity and induce browning of white fat, while undesirably induce weight gain, visceral obesity and other adverse effects. Thus, deciphering the combinatorial interactions between PPARγ and its transcriptional partners and their preferential regulatory network in the processes of development, function and senescence of adipocytes would provide us the molecular basis for developing novel partial agonists that promote benefits of PPARγ signaling without detrimental side effects. In this review, we discuss the dynamic components and precise regulatory mechanisms of the PPARγ-cofactors complexes in adipocytes, as well as perspectives in treating metabolic diseases via specific PPARγ signaling.

## Introduction

Adipose tissue is critical in maintaining energy homeostasis and insulin sensitivity in vertebrate organisms by directing energy fluxes toward triglyceride synthesis for storage or fuel mobilization for utilization in response to different metabolic requirements. Genetic, dietary, pathological, or aging-related disruption of adipose tissue function is one of the underlying causes of the current pandemics of metabolic diseases including obesity and type 2 diabetes. Adipose tissue exerts its normal physiological function via a complicated and delicate network of transcription factors to orchestrate and fine-tune various molecular events in adipocytes development, functionality, and senescence, among which peroxisome proliferator activated receptor γ (PPARγ) holds special importance for its unique and indispensable role in adipogenesis, lipid metabolism and insulin sensitivity (1). Moreover, evidences from murine and *Caenorhabditis elegans* models indicate that PPARγ is also a key driver in promoting longevity (2, 3). As a ligand dependent transcription factor, PPARγ exerts pleiotropic functions through its vast sets of downstream transcriptional targets. Mechanistic studies reveal that, under different stimuli or physiopathological status, PPARγ forms distinct transcription complex with different set of cofactors and transcription partners to regulate specific transcriptional circuit. Though the upstream regulators of PPARγ transcription is well studied and documented, from an empirical standpoint, it would be intriguing to decipher the detailed molecular events during which PPARγ integrates and transduces diverse signals toward specific clusters of downstream targets. In this review, we aim to discuss our current understanding of the combinatorial interactions between PPARγ and its interaction partners and how their dynamic changes impact PPARγ downstream target gene transactivation under different metabolic scenario and during the aging process in adipose tissues, thus potentially provide us with more elaborate and precise strategies to screen novel therapeutic targets that target PPARγ and its cofactors in treating adipocyte dysfunctions and metabolic diseases.

## Distinct roles of adipocytes in energy homeostasis

Three distinct types of fat (white, brown, and beige adipose tissue) exist based on different anatomic locations and functions (4). White adipocytes feature a single large lipid droplet and store excess energy in forms of triglycerides. In sharp contrast to white adipocytes, brown adipocytes, characteristically contain multiple lipid droplets and packed with mitochondrial, are vital in defending body temperature through activation of uncoupling protein 1 (UCP1) and uncoupling the mitochondrial respiratory chain to produce heat, thus contribute greatly for energy expenditure and adaptive thermogenesis. Interspersed within white fat, the newly discovered beige adipocytes share functional similarities with both white and brown adipocytes. Briefly speaking, beige adipocytes are indistinguishable from white adipocytes morphologically under basal condition. While upon cold or adrenogenic signaling stimulation, beige adipocytes are activated (referred to as the “browning of white fat” effect) and take on a brown adipocyte-like look with multiple small lipid droplets and rich mitochondria content. Similar to brown fat, activated beige adipocytes have high UCP1 expression and enhanced thermogenic capacity and energy expenditure (5–7). Importantly, upon activation, brown, and beige adipocytes uptake high levels of lipid and glucose for heat production, thus serve as a metabolic sink to clear excess nutrients in blood and contribute to insulin sensitivity and whole body lipid/glucose metabolism (8), in addition to the lowing body weight effect. Together, white, brown and beige adipocytes maintain the whole-body energy homeostasis.

Though rise from different precursors, white, brown and beige adipocytes all go through a finely tuned differentiation process (referred to as “adipogenesis”) to mature and become fully functional (9). Then, mature adipocytes can respond to different metabolic stimuli to store or utilize energy, as well as crosstalk with other cell types via various adipokines, cytokines or lipid/glucose fluxes (10). Eventually, adipocytes undergo senescence and gradually lose their differentiation or thermogenic capability (11, 12). During various stages of the adipocyte lifespan, PPARγ is a well-established central player in orchestrating the numerous molecular events that ensure the normal physiological function of white, brown and beige adipocytes. PPARγ, a member of the nuclear receptor PPARs family, shares the common PPAR domain structures that feature a highly conserved DNA-binding domain (DBD) and a transactivation domain (AF1) at the N-terminal region, and a ligand-binding domain (LBD) and a ligand-dependent transactivation domain (AF2) at the C-terminal region. PPARγ has two isoforms, PPARγ1 and PPARγ2, the latter contains an additional NH2-terminal region composed of 30 amino acids due to different promoter usage and alternative splicing (13). PPARγ1 expresses ubiquitously while PPARγ2 expresses the highest in adipose tissues and is highly inducible in other tissues under high fat diet (HFD) (14). By forming distinct transcription complexes with different interaction partners or through epigenetic modifications, PPARγ exerts critical and pleiotropic functions in adipose tissues that include: (I) adipocyte differentiation and lipid storage, (II) acquisition of brown/beige adipocyte identity (III) maintenance of brown/beige adipocyte thermogenic capacity, (IV) brown/beige adipocyte functional decline in aging, and (V) regulation of diabetic gene program (15). The dynamics beween PPARγ and its transcription partners in these physiological processes are discussed below.

### PPARγ/corepressors/coactivators dynamics controls adipocyte differentiation

PPARγ is considered the master regulator for adipogenesis since ectopic expression of PPARγ alone in fibroblasts could successfully drive the adipogenesis program and no other factors could induce adipogenesis without the presence of PPARγ (16–18). *In vivo* studies reveal PPARγ is vital in controlling adipogenesis and lipid/glucose metabolism. For instance, aP2- and adiponectin-driven loss of PPARγ in adipose tissues consistently lead to impaired adipocyte differentiation and reduced fat weights or lipodystrophy, though different animal models show improved or worsen insulin sensitivity depend on the extent of PPARγ deficiency (19–21). In adult mice, PPARγ ablation in adipose tissues with tamoxifen-dependent Cre-ER(T2) recombination system leads to adipocyte death and subsequent renewal, suggesting that PPARγ is required for the survival of mature adipocyte (22, 23). In clinic, patients with heterozygous PPARγ mutation show partial lipodystrophy and insulin resistance (24). PPARγ is also a critical thrifty gene that governs hoarding gene programs for energy storage mostly in white adipocytes (25).

Adipocytes differentiation is a closely regulated process that rises in response to physiological needs, i.e., adipocytes turnover and excess nutrient influx. Though it may function differently in aging (26), during development and in adulthood, PPARγ controls the transcriptional activation of the adipogenesis process through ligand binding and selective interaction with transcription corepressors or coactivators (17). Corepressors bind to transcription factor/DNA complexes to recruit histone deacetylases (HDAC) and make the target DNA region less accessible for transcription. On the contrary, coactivators possess intrinsic histone acetyltransferase (HAT) activity or recruit other proteins with HAT activity to acetylate histones and loosen the chromatin in a limited region, allowing for increased basal transcription machinery accessibility (27). PPARγ constitutively forms a heterodimer with retinoid X receptor (RXR) and binds to a specific DNA sequence termed PPAR response elements (PPRE). In basal condition, unliganded PPARγ forms a complex with transcription corepressors such as silencing mediator of retinoid and thyroid hormone receptors (SMRT), the nuclear receptor corepressor (NCoR), and HDACs, which blocks its transactivation activity. Upon ligand binding, PPARγ undergoes a conformational change, dissociates corepressors and recruits coactivators i.e., CREB-binding protein (CBP), histone acetyltransferase p300 (p300) and PPAR-binding protein (PBP), thus initiates the adipogenic gene programs including Fatty Acid Binding Protein 4 (Fabp4), Cluster of differentiation 36 (Cd36), Adiponectin, Fatty Acid Synthase (Fasn), etc. (28, 29). It has been shown that coactivators deficiency in fibroblasts hinders adipocyte differentiation while adipocytes lack corepressors accumulate more lipids, which demonstrates the dynamic competition between activating vs. suppressive PPARγ-cofactors transcriptional complexes in adipogenesis (30, 31).

### PPARγ/PRDM16/EBF2/EHMT1 complex determines brown/beige adipocyte identity

It is demonstrated that brown adipocytes and skeletal muscle cells rise from the common *Myf5*+ Dermomyotomal precursors while beige adipocytes and white adipocytes share the common *Pdgfra*+ mesodermal stem cells (32). Besides its indispensable role in promoting differentiation of white, brown and beige adipocytes, another critical function of PPARγ resides in the determination of brown and beige adipocyte identity. To achieve this, PPARγ recruits PRDM16, EBF2, and EHMT1 to coordinate the transcriptional circuits toward the brown/beige lineage (33).

For brown/beige adipocytes to acquire their identity and become thermogenic poised adipocytes, PPARγ recruits PR (PRD1-BF1-RIZ1 homologous)-domain-containing 16 (PRDM16) to form a core transcription complex that determines the transition of brown adipocyte from skeletal muscle cell or beige adipocyte from white adipocyte. Mass spectrometry analysis indicates PPARγ as the only DNA binding transcription factor interacts with PRDM16 in a near stoichiometric manner (34). This is further proved when full length GST-PPARγ2 fusion protein binds to *in vitro* translated PRDM16. PRDM16 is a powerful inducer and maintainer of the thermogenic phenotype in both brown adipocyte development and the browning process (35, 36). Mechanistic studies show that PRDM16 binds to PPREs on the promoter and/or enhancer of brown fat-selective genes and highly stimulates the activity of a PPRE luciferase reporter. Besides, PRDM16 fails to promote adipogenesis in PPARγ-deficient fibroblasts (37), indicating it at least partially exerts function via PPARγ. In myocytes, in the presence of PPARγ agonists, PRDM16 induces multiple PPARγ target genes in adipogenesis like aP2 and adiponectin, as well as brown fat-selective gene program, including Ucp1 and Cidea. Later, it is revealed that PPARγ agonists could induce a white-to-brown fat conversion through stabilization of PRDM16 protein, suggesting the existence of a positive regulatory loop to strengthen the interaction of the PPARγ/PRDM16 complex to maintain the thermogenic capacity in beige adipocytes (38). *In vivo* studies echo the results from *in vitro* studies. Transgenic and tissue specific animal models show that increased expression of PRDM16 in rodent inguinal white adipose tissue (iWAT) and in mature adipocytes promotes beige adipocyte commitment and prevents HFD-provoked weight gain and systemic insulin resistance (39). Conversely, deletion of Prdm16 in adipocytes causes a profound loss of beige adipocytes in mice, leading to aggravated metabolic deregulation upon exposure to HFD (40).

Two other factors, Histone-lysine N-methyltransferase (EHMT1) and Early B-cell factor (EBF2), present in the PPARγ/PRDM16 complex and facilitate its function in brown/beige fat identity determination (41). EHMT1 is the only methyltransferase purified specifically with PRDM16 by mass spectrum. It induces the inhibitory H3K9me2 and H3K9me3 at PRDM16-resident myogenic gene promoter regions, thus enables the PPARγ/PRDM16/EHMT1 to promote the common Myf5^+^ precursors toward brown adipocytes linage instead of muscle linage. EHMT1 also controls thermogenesis by stabilizing PRDM16 and extending its half-life. Similar to PRDM16, EHMT1 fat-specific deficient mice show obesity and insulin resistance (41). Moreover, it has been reported that activation of brown adipocytes in the adult human perirenal depot is highly correlated with PRDM16–EHMT1 complex expression (42).

On the other hand, it is demonstrated that PPARγ recruits EBF2 to its brown-selective binding site and coactivates the expression of brown fat-selective genes such as Ucp1, Pparα, and Prdm16 (43). Genome-wide ChIP-sequencing and motif analyses of the consensus binding site for EBF2 reveal that it is highly enriched in brown adipocytes compared to white and beige fat. Consistent with its function in coactivating PPARγ downstream brown fat-selective gene program, EBF2 deficient mice fail to thrive due to severe defects in brown fat development and themogenesis (43). Intriguingly, transcription regulator ZFP423 maintains white fat identify via inhibition of EBF2 and PRDM16 activity (44), further suggesting the critical role of PPARγ/PRDM16/EBF2 in brown adipocyte development.

Overall, the PPARγ/PRDM16/EBF2/EHMT1 complex determines the identity of brown/beige adipocytes. It has been shown that the loss of brown and beige fat may predispose the obese and aging population to metabolic dysfunction, thus future extensive study of this complex is fundamental for developing compounds that could drive precursor cells to undergo brown/beige adipocytes fate and potentially achieve enhanced thermogenesis and energy expenditure.

### PPARγ/PRDM16/PGC1α modulates brown/beige adipocyte functionality

After the thermogenic endowment of brown/beige fat, the PPARγ/PRDM16 complex recruits another distinct set of cofactors to promote brown/beige fat function in adaptive thermogenesis and energy homeostasis, among which PPARγ-coactivator PGC1α plays a central role. PGC1α is first cloned from murine brown fat cell cDNA library through yeast two-hybrid system using PPARγ partial protein as bait (45). As a coactivator, PGC1α is capable of binding to and coactivating many nuclear receptors and transcription factors to exhibit different functions, such as PPARα, β/δ for fatty acid β-oxidation, thyroid receptor and IRF4 for thermogenesis, HSF1 for heat shock responses, NRF1 and NRF2 for mitochondrial biogenesis (46–48). In brown fat, PGC1α promotes mitochondrial biogenesis and thermogenesis at least partially by coactivating PPARγ and enhancing PPARγ's transcription activity on the thermogenic gene program, including Cidea, Elovl3, and Ucp1 (49). Moreover, it has been found that PRDM16 directly interacts with PGC1α, which increases PGC1α expression and promotes PGC1α transactivation activity (50). These data suggest the existence of a PPARγ/PRDM16//PGC1α thermogenic transcription complex in the regulation of brown/beige functionality. This core complex in turn recruits other cofactors or undergoes various modifications to fine tune thermogenesis and energy homeostasis.

For example, MED1 is found to be enriched along with PPARγ/PRDM16 complex on the promoters of brown fat-selective genes (51). As a component of mediator complex, MED1 plays a crucial role in regulating transcription in part through bridging the transcription factor-bound enhancer regions with the general transcriptional machinery and RNA Pol II at gene promoters. In the brown/beige adipocytes context, MED1 physically binds to PRDM16 and is then recruited to super enhancers at brown fat-selective genes. Consistently, MED1 binding at PRDM16 target sites reduces in PRDM16 deficiency BAT, causing a fundamental change in chromatin architecture at key brown fat-selective genes (52).

The SRCs family, also known as p160 proteins, includes SRC-1/NCoA-1, SRC-2/GRIP1/TIF2/NCoA2, and SRC-3/p/CIP/AIB1. They are transcriptional coactivators that interact with nuclear receptors and enhance their transactivation in a ligand-dependent manner (27, 53). In the metabolic context, they work as cofactors for PPARγ/PGC1α to influence brown/beige function and energy homeostasis. For Instance, SRC-1^−/−^ mice showed partially impaired PPARγ function in the brown fat and are prone to obesity due to reduced energy expenditure and fatty acid oxidation upon HFD feeding (54, 55). Mechanistic study reveals that SRC-1 stabilizes PPARγ/PGC1α interaction and thus increases PGC1α-mediated adaptive thermogenesis (54). On the contrary, SRC-2^−/−^ mice are protected against obesity. In white fat, PPARγ preferentially recruits SRC-2 as its coactivator to transactivate downstream fat uptake and storage pathways while in the brown fat SRC-2 competes with SRC-1 for PPARγ binding and disrupts SRC-1-induced interaction between PPARγ and PGC1α (56, 57). Meanwhile, in SRC-3^−/−^ mice, HFD feeding causes a depot-selective decrease in PPARγ2 level, resulting in body weight reduction, fat mass decrease and fat redistribution in knockout mice (58, 59). Besides, SRC-3 induces PGC1α acetylation and consequently inhibits its activity in brown fat (60). SRC-3 also cooperates with SRC-2 to attenuate PPARγ phosphorylation at S114, which in turn increase PPARγ transcriptional activity and adipogenesis (61). Collectively, these data summarize the crucial roles of SRC family members that cooperate or antagonize with each other to regulate the interaction of PPARγ/PGC1α complex and its transactivation activity on the thermogenic network.

The stability of PPARγ/PRDM16 complex also impacts the induction of brown fat gene programs and thus the brown/beige fat function. It is well demonstrated that SirT1-dependent deacetylation of PPARγ Lys268 and Lys293 facilitate the close interaction between PPARγ and PRDM16 and is essential for the selective induction of brown fat-selective genes and repression of visceral white fat-selective genes (62). Silencing SIRT1 in 3T3-L1 preadipocytes leads to their hyperplasia and increased expression of white fat and inflammatory markers with a parallel decrease in brown fat markers (63). Specific genetic ablation of SIRT1 in WAT leads to obesity, increased inflammatory infiltration, and insulin resistance similar to that observed in HFD induced obesity, further suggesting SIRT1 deficiency leads to whitening effects of mature adipocytes (64). Conversely, SirT1 pharmacologic activators, i.e., resveratrol is shown to be effective in inducing the browning of white fat, weight loss, and improved insulin sensitivity in mice, though possibly through multiple mechanisms in addition to SirT1 activation (65). On the other hand, a few negative regulators of browning work through disrupting PPARγ/PRDM16 interactions. For example, TLE3 is a white-selective cofactor that competes with PRDM16 for PPARγ interaction to specify lipid storage or thermogenic gene programs (66). In addition to transcriptional factors and cofactors, PexRAP, a peroxisomal lipid synthetic enzyme interacts with PPARγ and PRDM16 and disrupts PRDM16-mediated gene expression (67). Last but not least, it has to be noted that nuclear factor I-A (NFIA) is recently reported to co-localize with PPARγ at the brown fat specific enhancers and facilitate PPARγ chromatin accessibility for induction of brown fat gene programs through mechanisms independent of the PPARγ/PRDM16 complex (68).

Collectively, these findings extend our understanding on how the PPARγ/PRDM16/PGC1α thermogenic transcriptional complex ensures delicate regulation on brown/beige fat functions by fine tuning its various components and modification status. The fact that the thermogenic transcriptional complex is required for brown/beige adipocytes to be fully functional supports the notion of targeting it for future potential drug discoveries to treat obesity and metabolic diseases, while inhibition of the negative regulators of this complex provides alternative strategies of inducing browning for energy expenditure.

### PPARγ/cofactors complex in aging

Life expectancy increases with the advancement in modern medicine and the improvement in life quality, bringing forward an aging society. It has been well accepted that energy homeostasis is tightly controlled by the central nervous system, most prominently in the hypothalamus and brainstem areas that receive and integrate peripheral signals such as insulin and leptin, and in turn regulate food intake and energy metabolism in peripheral organs including adipose tissues, livers, and muscle (69). Thus it is not surprising that metabolic dysfunction occurs in line with aging due to the gradual deterioration in brain function. In both rodents and humans, advanced aging features significant weight loss due to anhedonia, anorexia, lipodystrophy, and muscle atrophy caused by the deregulations in brain (70). Intriguingly, during aging, peripheral organs such as adipose tissues undergo programmed functional loss and senescence as well. It is well documented that during aging, adipose tissues undergo a gradual exhaustion in progenitor pool, impaired proliferation and differentiation capability in preadipocytes and programmed functional loss in mature adipocytes (71, 72), resulting in disrupted lipid and glucose metabolism and impaired energy homeostasis. The aging trajectory begins around middle age after the peak fertility is passed. Compared to the phase of advanced aging, this phase often features an increase in body weight and fat mass, impaired metabolic fitness, and deregulated lipid profiles, during which the senescence and dysfunction of adipose tissues play a profound role. Indeed, midlife weight gain is one of the major risk factors for metabolic syndrome such as Type 2 diabetes, cardiovascular diseases, hypertension, hyperlipidemia, hepatic steatosis, and certain types of cancer, which poses a serious burden on public health management nowadays (73, 74). Thus, how aging affects adipocyte function and its intrinsic mechanism attract great attentions in the recent years (75).

As the master regulator in adipocytes, with the triumph of specific cre/loxP recombination system, the *in vivo* roles of PPARγ and its cofactors in fat biology during development or in young adult animals have been extensively studied. However, the existing aP2-Cre/loxP or adiponectin-Cre/loxP system deletes gene in fat tissues in early stages of life, the results of which are hard to extrapolate to the aging process. It has been shown that the PPARγ agonist rosiglitazone (TZD) treatment rescues shortened lifespan in *C. elegans* and induces a transcriptional signature that overlaps with longevity-associated genes (3). Besides, hypomorphic PPARγ mice and PPARγ2 heterozygous mice both show reduced lifespan (2), indicating an important role of PPARγ in aging. Considering the metabolic derangement in adipose tissues during aging and the critical role of PPARγ in adipocytes, it merits further investigation on the dynamics of PPARγ and its cofactors specifically in the aging process and how they impact the functional decline in adipose tissues.

**Figure 1:**
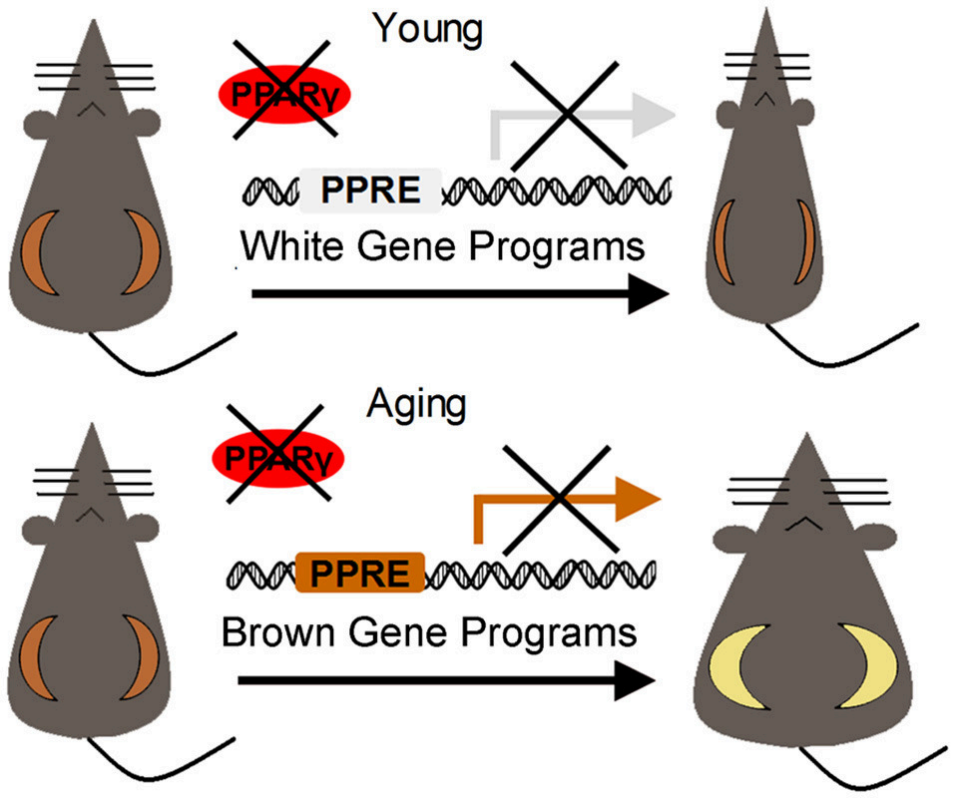
Preferential regulation of white and brown gene programs by PPARγ in beige fat during aging.

Using the adenoviral delivery strategy, Ma et al. reported the surprising finding that loss of PPARγ specifically in subcutaneous fat in aging mice (12-month-old) is sufficient to increase body weight and insulin resistance by accelerating the decay of browning effects of white fat and disrupting energy homeostasis. Detailed analysis reveals that in aging adipose tissues, PPARγ preferentially binds to the promoters of browning vs. whitening gene program, thus maintains browning capability of subcutaneous fat of aging mice rather than lipid storage, as shown by gene program expression array and *in vivo* ChIP analysis (Figure 1). Meanwhile, although the total PPARγ levels do not change, PPARγ Ser273 phosphorylation increases during aging, which may potentially affect PPARγ affinity with its cofactors and rewire its metabolic circuitry toward brown gene program (26). Additionally, in rodent and human adipose tissues, SRC-1 expression levels decrease during aging, which may disrupt PPARγ/SRC-1 complex and its function in insulin sensitivity and adipogenic activity (76). These works offer novel insights on the unique combinatorial organization of the PPARγ/cofactors complex and the resulting target gene sets under the aging scenario, which might be largely different than previously reported PPARγ function in young animal models. From a therapeutic point of view, these results emphasize the need of personalized treatment when targeting PPARγ to treat metabolic disorders in patients of different ages. It would also be of importance to further investigate the potential benefits of targeting PPARγ in preventing age-associated metabolic diseases and promoting longevity.

### PPARγ phosphorylation in diabetic/adipogenic/osteogenic gene program

In addition to recruitment of different cofactors, the post-translational regulations of PPARγ add another layer of regulation for the PPARγ/cofactors complex to selectively transactivate different gene circuits. In clinic, the full PPARγ agonists TZDs are very effective in glycemic control but have multiple adverse effects such as obesity, water retention, and increased risk of cardiovascular diseases and bone fractions. The opportunity for improvement emerges when Spiegelman's lab identifies the distinguish role of PPARγ Ser273 phosphorylation in lipid metabolism and insulin sensitivity (77–79). Briefly, in HFD induced obesity, protein kinase cyclin-dependent kinase 5 (Cdk5) is activated and in turn phosphorylates PPARγ at Ser273. This form of post-translational modified PPARγ selectively transactivates target genes involved in insulin resistance, without significant changes in adipogenic or osteogenic gene program (77). Consistently, Ma et al. report increased PPARγ Ser273 phosphorylation in iWAT of 12-month-old mice, which may contribute to impaired insulin sensitivity during aging (26).

PPARγ Ser273 phosphorylation is regulated by its interacting cofactors, which specifies a regulatory role of PPARγ/cofactors complex on the diabetic gene program. For example, PPARγ corepressor NcoR functions as an adaptor to enhance Cdk5 activity on interacting and phosphorylating PPARγ Ser273. Consistently, when fed a HFD, compared to wild type mice, fat-specific NCoR-deficient mice are prone to obesity yet have enhanced insulin sensitivity (80). Upon PPARγ Ser273 phosphorylation, phosphorylated PPARγ recruits thyroid hormone receptor-associated protein 3 (thrap3) to facilitate the expression of the diabetic gene programs (78). These data establish a delicate control of diabetic genes by PPARγ/cofactor complex through PPARγ Ser273 phosphorylation, as well as the potential of targeting PPARγ Ser273 phosphorylation to improve insulin sensitivity while eliminating the disadvantage of side effects. Of therapeutic significance, ERK have been shown to phosphorylate PPARγ Ser273 and induce insulin resistance in the absence of Cdk5, rendering ERK as promising anti-diabetic targets (79). Moreover, novel PPARγ partial ligands such as SR1664, F12016, and antibiotic ionomycin are designed or screened to inhibit PPARγ Ser273 phosphorylation, which show effective glycemic control with minimal side effects (81–83).

Aside from its numerous functions in adipocytes, PPARγ also plays vital role in controlling the balance between bone marrow adipogenesis and bone formation. For instance, PPARγ phosphorylation at Ser112 by mitogen-activated protein kinases (MAPKs) inhibits adipogenesis while promoting osteogenesis (84). A non-phosphorylatable Ser112 PPARγ mutation drives adipogenic differentiation and inhibited osteoblastogenesis *in vitro*. Consistently, mice carrying this homozygous PPARγ mutation (PPARγ-S112A mice) show increased bone marrow adipose tissue with reduced bone volume. In another study, PPARγ phosphorylation at different sites exhibits osteoblastic (pS112) or osteoclastic (pS273) activity thus regulates bone formation and absorption in a delicate balance. These collective data, along with the role of PPARγ Ser273 phosphorylation in promoting diabetic gene program, show the advantage of using PPARγ agonists against selective phosphorylation sites. For instance, SR10171, a PPARγ S273 phosphorylation blocker, increases both insulin sensitivity and osteoclastogenesis (85) while PPARγ partial agonist Telmisartan, which decreases PPARγ S273 without altering pS112 phosphorylation, promotes browning effects and improves insulin sensitivity without altering bone mass or bone biomechanical properties in mice (86). Thus, it would be desirable to develop pharmacologic agents targeting specific PPARγ phosphorylation sites, especially ones that dephosphorylate S273 while maintaining pS112 phosphorylation, to maximize their beneficial activities.

## Perspectives

In clinic, PPARγ Pro12Ala polymorphism is identified as one of the common risk polymorphisms for Type 2 diabetes in multiple GWAS studies (87). Indeed, the thiazolidinedione class of drugs, the PPARγ full agonists, has a long history of use in treating Type 2 diabetes mellitus for its insulin sensitizing effects, though multiple side effects exist including obesity, water retention, increased risk of cardiovascular diseases and bone fractions. Besides, recent studies indicate PPARγ play an important role in antiretroviral treatment related adipocyte dysfunction and lipodystrophy in HIV-infected patients. These studies potentiate the importance of developing PPARγ partial agonists to selectively activate a specific set of its numerous target genes in order to avoiding possible side effects. To date, various PPARγ partial agonists are in different stages of drug development yet none has progressed into clinic due to insufficient efficacy and potential adverse effects, which merits a close examination to gain more mechanistic insights of PPARγ.

**Figure 2:**
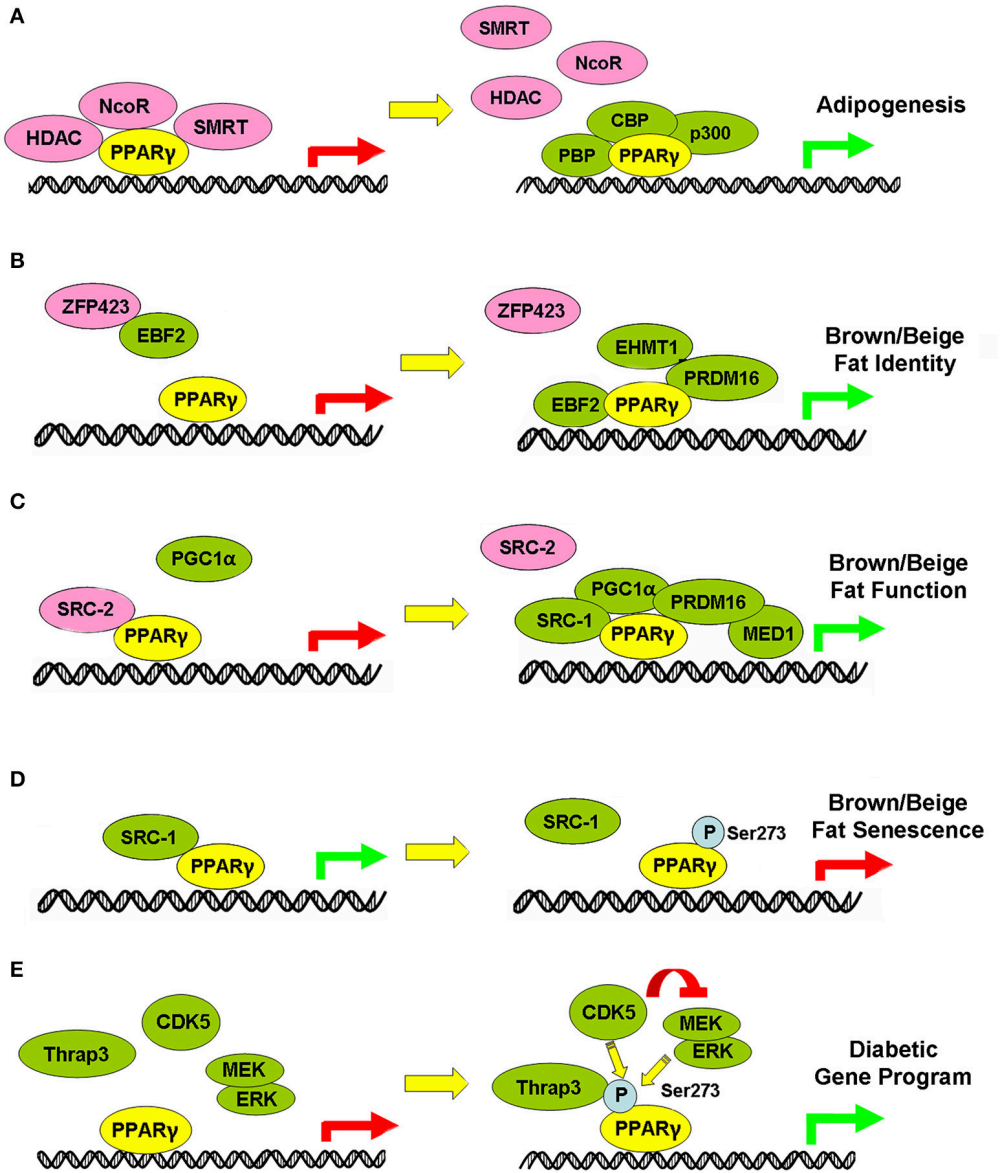
Illustration of dynamic regulation of PPARγ-centered complex in different physiological processes of adipocytes. Green arrows indicate activation, while red arrows indicate suppression of transcriptional gene programs. Green and Pink circle indicates proteins in cooperation with PPARγ to activate and suppress specific gene programs. **(A-E)** PPARγ-centered complex in the regulation of Adipogenesis **(A)**, Brown/beige fat identity **(B)**, Brown/beige fat function **(C)**, Brown/beige fat senescence **(D)** and Diabetic gene program **(E)**.

Of note, when developing PPARγ agonists, it is necessary to put the interaction between PPARγ and its cofactors into the equation since after ligand binding, PPARγ recruits transcription partners and cofactors to form a transcription complex to achieve selective target gene transactivation. In this review, we highlight a comprehensive review of the dynamics in transcription partners, coregulators, and post-translational modifications of PPARγ in adipose tissues during various stages of the adipocyte life cycle and the resulting regulatory function on fat development, thermogenesis and adipocyte senescence (Figure 2). PPARγ dissociates corepressors (e.g., NCoR, HDAC, SMRT) and binds with coactivators including CPB, p300, and PBP to initiate adipocyte differentiation in white, brown and beige adipocytes. In brown and beige adipocytes, PPARγ complexes with PRDM16/EBF2/EHMT1 for brown/beige cell linage determination while their unique functions in adaptive thermogenesis and energy homeostasis are maintained and modulated by the PPARγ/PRDM16//PGC1α complex as well as their interaction with various cofactors i.e., MED1, SRC1/2/3, and TLE3. Of therapeutic relevance, under aging scenario, PPARγ may complex with different cofactors due to different post-translational modifications or cofactor expression levels and thus exert distinct function as compared to its function in young animals. Last but not least, modulation of phosphorylation status of PPARγ selectively activates the diabetic or adipogenic circuits in adipocytes, which serves as an attractive strategy for therapeutic development. In recent years, the triumph of high-throughput sequencing technology has offered us the whole picture of the binding profiles of PPARγ on the genome-wide scale in white, brown and beige adipocytes under different metabolic conditions, which enable us to gain a deeper understanding of the common and distinct target gene sets regulated by PPARγ in different fat depots, under different diet regimes or ages, or in response to different environmental or drug stimulus. However, challenge remains as how PPARγ achieves differential regulatory patterns in different scenarios. To achieve this, multiple ChIP sequence analyses using various PPARγ cofactors are needed to link the dynamics of PPARγ and its transcription partners with specific transactivation circuits and functionality.

During gene transcription, upon transcription factor binding, cofactors add an extra layer of transcriptional regulation on target genes and endow enhanced regulatory complexity. To combat the high prevalence of metabolic diseases worldwide, the needs for novel PPARγ agonists that feature maximum beneficial effects i.e., efficient glycemic control or enhanced “browning” effects while avoid undesirable side effects are heightened. With the crystal structure of PPARγ fully resolved and the accumulating data on its conformational changes upon full or partial agonists binding, a large body of works focuses on finding potential novel PPARγ agonists via computational predictions and virtual screening strategies. However, despite binding to the PPARγ ligand binding domain with high affinity as predicted, these compounds often face the problem of low transactivation activity *in vitro*, which hinders their progression into clinic. In this regard, the importance of cofactors during PPARγ transactivation events is underappreciated. It would be more informative to resolve the structure changes of PPARγ in the presence of various cofactors i.e., PRDM16 and PGC1α and take the dynamics between PPARγ and its cofactors into consideration when designing new PPARγ agonists in the future. For example, strategies could be developed to screen for agonists that could strengthen the interaction between PPARγ and specific coactivators or block PPARγ phosphorylation at specific sites.

In previous reports, specific PPARγ deletion in adipose tissues in young adult animals results in lipodystrophy or massive adipocyte death, indicting an indispensable role of PPARγ in adipose tissue development and survival. Unexpectedly, PPARγ deletion in a temporal-specific manner in inguinal fat of aging animals causes significant increases in body weight and fat mass, which is in sharp contrast to young control animals (26). Further analyses show PPARγ has unique spectrums of interaction partners and target genes in aging, which is in consistent with previous reports of PPARγ as a potential driver for increased longevity (2, 3, 26). These findings shed first light on the potential of using PPARγ agonists in aged population to promote healthspan and lifespan. As a transcription factor with pleiotropic functions, targeting PPARγ could have multiple benefits against various aspects of aging. Firstly, aging features metabolic derangement, toward which PPARγ agonists show high efficacy in glycemic control, thus promoting metabolic fitness during aging. Secondly, caloric restriction (CR) is a well-established nutritional intervention that increases longevity and promotes metabolic health in various species from *C. elegance* to primates (88). It is reported that PPARγ expression levels are dramatically induced by CR treatment in metabolic organs, especially in inguinal fat for browning of white fat, suggesting that PPARγ activation might mimic CR effects at least in fat tissues (89). Thirdly, recent reports show compounds classically functioning through glycemic control (i.e., metformin) or immunosuppression (i.e., rapamycin) are effective in promoting longevity (90, 91). Considering the dual function of PPARγ in glucose metabolism and inflammation control, it would be interesting to test its agonists in longevity.

Overall, although PPARγ has been recognized as a promising target in preventing and treating metabolic diseases, challenges remain in finding the suitable agonists with high therapeutic efficacy and low side effects. Understanding how PPARγ dynamically complexes with its cofactors to transactivating specific downstream target gene sets in different metabolic conditions might provide a novel angle in search of new therapeutic compounds against various aspects of obese- and aging-associated metabolic diseases.

## Author contributions

XM and LX conceived, drafted and revised the review. DW and WZ drafted the review.

### Conflict of interest statement

The authors declare that the research was conducted in the absence of any commercial or financial relationships that could be construed as a potential conflict of interest.
